# Supplementary material for: Bayes Forest: a data-intensive generator of morphological tree clones
Source: Gigascience. 2017 Aug 19;6(10):1–13. doi: 10.1093/gigascience/gix079 (PMC5632294; doi:10.1093/gigascience/gix079)

Manuscript with DOI
Click here to download Manuscript MorphoClones_Giga_rev1_WITH DOI.docx

Bayes Forest: a data-intensive generator of morphological tree clones

Ilya Potapov[1], Marko Järvenpää[2], Markku Åkerblom[1], Pasi Raumonen[1], Mikko Kaasalainen[1]

[1]Mathematics Department, Tampere University of Technology, P.O. Box 553, 33101, Tampere, Finland

[2]Helsinki Institute for Information Technology, Department of Computer Science, Aalto University, Finland

Emails (in the author order, * - corresponding author):

* ilya.potapov@tut.fi, marko.j.jarvenpaa@aalto.fi, markku.akerblom@tut.fi, pasi.raumonen@tut.fi, mikko.kaasalainen@tut.fi

Abstract.

Background. Detailed and realistic tree form generators have numerous applications in ecology and forestry. For example, varying morphology of trees contribute differently to formation of landscapes, natural habitats of species, and eco-physiological characteristics of the biosphere.

Findings. Here, we present an algorithm for generating morphological tree "clones" based on the detailed reconstruction of the laser scanning data, statistical measure of similarity, and a plant growth model with simple stochastic rules. The algorithm is designed to produce tree forms, i.e. morphological clones, similar (and not identical) in respect of tree-level structure, but varying in fine-scale structural detail. Although we opted for certain choices in our algorithm, individual parts may vary depending on the application, making it a general adaptable pipeline. Namely, we showed that a specific multi-purpose procedural stochastic growth model can be algorithmically adjusted to produce the morphological clones replicated from the target, experimentally measured tree. For this, we developed a statistical measure of similarity (structural distance) between any given pair of trees, which allows for the comprehensive comparing of the tree morphologies by means of empirical

distributions describing geometrical and topological features of a tree. Finally, we developed a programmable interface to manipulate data required by the algorithm.

Conclusions. Our algorithm can be used in a variety of applications for exploration of the morphological potential of the growth models (both theoretical and experimental), arising in all sectors of plant science research.

Keywords: quantitative structure tree model; morphological clone; stochastic data driven model; terrestrial laser scanning; large scale data; empirical distributions

Findings

I. Background

Models for plant architecture attract significant attention due to their ability to assist the empirical studies in ecology, plant biology, forestry, and agronomy [1]. The modeling activity is especially useful in research since it arises as fruitful collaboration between specialists in different fields of studies: computer scientists, mathematicians, and biologists [2].

Modeling plant architecture is approached from many directions. Some progress has been achieved in synthesis of realistic plant forms in the field of computer graphics [3-5]. These models, although based on heuristic rules of growth, produce realistic tree representation in a fast and efficient manner, which is usually dictated by the application of this approach, i.e. natural scene in computer visualization. Heuristic growth rules of the procedural models for graphics applications are not firmly based on

biological principles, but nevertheless elucidate some algorithmic properties of the growth process (for example, recursive [6] vs. self-organizing [3, 7] character of architecture development).

However, the most promising plant architectural models are so called functional-structural plant models (FSPM) [8-10], because this type of models allows for a balanced description between morphological and functional/physiological properties of a plant. It is capable of connecting the external abiotic factors (e.g. radiation, temperature and soil) and the most vital functions of a plant organism (such as photosynthesis, respiration, and water and salts uptake) with its structural characteristics [1, 2].

Nevertheless, biologically relevant architectural plant models rely on data in a form of empirically fitted functions and parameters that correspond to a particular species and/or certain site conditions [11-14]. Thus, the change in these conditions requires re-calibration of the models, which is done in a manual fashion every time the model is simulated for the new conditions. Strong dependence on data, where each simulation would be calibrated automatically by data, is limited by both computation time and lack of the fast measurement and processing systems allowing for a detailed 3D morphological reconstruction of the actual plant/tree.

The most recent advances in laser scanning techniques allow for fast and non-destructive measurement of trees with subsequent reconstruction of various characteristics depending on application (e.g. [15, 16]). Most of such studies dedicated to reconstruction of 3D point clouds obtained from laser scanning measurements deal with overall characteristics, such as height, width, and volume of stems/crowns, leaf index, biomass etc., resembling traditional destructive methods of measurement [15, 17]. However, the detailed precise geometrical and topological reconstruction of the tree architecture is never achieved perfectly.

We use a fast, precise, automatic, and comprehensive reconstruction algorithm initially presented in [18] and further developed and tested in [19]. The algorithm reliably reconstructs a quantitative structure model (QSM), which contains all geometrical and topological characteristics of the object tree. Input for the method is the 3D point cloud, sufficiently covering the tree, obtained from the terrestrial laser scanning measurements (TLS). No additional allometric relations used for estimation of the branch proportions (as in [20, 21]) are needed. Compared to other similar techniques (e.g. [20-22]) this method requires few parameters and no user interaction. It reconstructs the tree surface with subsequent cylinder (or any other geometrical primitive) approximation, which is usually consistent with theoretical plant growth models. The reconstruction algorithm has been validated in several studies with several different tree species and different scanner instruments [19, 23-26]. There are other published QSM reconstruction methods from TLS data that can produce similar quality QSMs, at least [23].

In this work, we utilize an inverse iterative procedure to optimize model's parameters for matching the (empirical) distribution of structural features of the simulated stochastic tree models (FSPM, graphical or other) to the tree reconstructed from the laser scanning data. Meanwhile, we formulate a measure of similarity of the tree structures based on the tomographic analysis of the structural distributions (e.g. Radon transform) [27, 28]. Finally, the optimal parameter set produces morphological "clone" trees with similar overall structure, but with varying fine-scale details.

Recently, we have reported a proof-of-concept study where we used reconstruction of a pine tree and the corresponding FSPM (named LIGNUM [13, 29]) to demonstrate the practical feasibility of the approach [30]. Here, however, we develop a unifying interface (in the form of a programmable toolbox) for our procedure and use general-purpose fast procedural tree growth model from [3]. This

procedural model is easier to adapt for technical experimentation with the whole algorithm. Similar algorithmic pipeline was reported in [5] for procedural tree growth models in the context of graphics synthesis. However, in our approach we see the tree growth as a random process and, consequently, apply corresponding statistical methods for measuring the similarity between trees. Moreover, in our algorithm we put emphasis on biologically and physically relevant descriptions, hence, the careful choice of the reconstruction algorithm. Another advantage is a possibility to use FSPM to relate physiological parameters to the morphogenetic processes in trees. Finally, we use no extra structures improving visual properties of trees but not supported by empirical observation (e.g. leaves). We note that any other choices of parameters and feature descriptions can be used in our approach, further facilitated with a programmable interface.

II. Algorithm overview

Our approach is based upon five distinct parts:

1. *Quantitative Structure Model (QSM)* is a reconstruction of a tree model from 3D point clouds obtained from terrestrial laser scanning measurements (TLS). Here we use specific algorithm for such reconstruction reported in [18] and [19] but other approaches could be used as well.
2. *Stochastic Structure Model (SSM)* is a tree growth model that is selected depending on the application. There are no limitations on the class of the model, except it must produce a measurable 3D branching structure.
3. *Structural data set (U)* is a collection of structural features (empirical distributions) to be compared between QSM and SSM. It is important that *U* be defined in the same way by both the QSM and SSM.

4. *Measure of structural dissimilarity, or structural distance D_S,* is a measure of discrepancy between any two data sets. In other words, $D_S(U_1, U_2)$ returns a value quantifying how much different the two data sets U_1 and U_2 are.
5. *Optimization algorithm* is a numerical routine capable of finding a minimum of any given function by varying its arguments. Examples include Newton algorithm, genetic algorithm, and simulated annealing.

The connection between these components is outlined in Fig. 1 with explanation in the text below.

Figure 1: The algorithm outline (see explanation in the text).

The algorithm outline (Fig. 1):

<u>Stage A: Preparation</u>

A1: build QSM from TLS.

A2: extract U_d from QSM.

<u>Stage B: Main cycle</u>

B1: simulate SSM (with fixed random generator seed for reproducibility) for the given parameters and extract U_m.

B2: compare U_m and U_d getting an estimation of the distance D_S between them.

B3: change SSM parameters trying to decrease D_S, go to B1 or stop and go to B4 (changing of the parameters and stopping criteria depend on any particular realization of the optimization routine).

B4: simulate SSM with the "best-fit" parameter values corresponding to the smallest found D_S.

B5: generate morphological clones using the best-fit SSM with different random number sequences.

At the preparation stage, the QSM is formed from the TLS point cloud (A1). The detailed description of this process is reported in [18, 19]. The resultant QSM contains all geometrical and topological features needed to form the empirical distributions U_d. The distributions can be formed from several tree individuals if they are close by shape to ensure the sample size of the data sets (e.g. a tree has a single main stem/trunk, hence its features are underrepresented).

At the main cycle of the algorithm, the empirical distribution U_m is formed from the simulated SSM tree (B1). Next, U_m is compared against U_d using the measure of distance (B2). The optimization routine iteratively minimizes the distance value every time changing the parameter values of SSM (B3), simulating SSM, and repeating the cycle from B1. After the stopping criteria of the optimization routine (number of iterations, minimal allowed distance etc.) are met, the algorithm stops and produces the best-fit SSM tree (B4). The best-fit SSM with different random sequences produces different morphological clones.

Below we describe general aspects of each of the main components of the algorithm. The Methods section addresses further the technical details.

Quantitative Structure Model (QSM)

QSM is derived from the point cloud obtained by TLS. Essentially, QSM is a surface reconstruction of the branches of the real tree measured by TLS. The reconstruction itself is a stochastic process, giving different architecture results for different runs. Therefore, the reconstruction introduces internal errors in addition to the TLS measurement errors. Besides giving spatial locations of parts of the tree, QSM also reconstructs topological relations between the tree branches. The branches of QSM consist of

elementary units, i.e. circular cylinders, but other geometrical primitives can also be applicable [31]. Thus, any potential structural information about the original tree can be approximated with high accuracy with QSM. The details of the reconstruction algorithm are presented in [18, 19], while the validation of the algorithm is show in [19, 23-25]).

Figure 2: The target QSM structure in three main 2D projections.

In this work, we use the reconstructed QSM of a maple tree (Fig. 2). The QSM was selected due to its non-trivial form and obvious irregularities in the tree growth. This is needed to determine whether the stochastic rules of SSM growth can account for this variability. In fact, the QSM growth irregularities might come from some deterministic sources, like constant wind, shading from the neighbors, animal influences etc. Thus, our algorithm tries to compensate the lack of knowledge of the growth process with simple stochastic rules of SSM and optimization of the stochastic distance function.

Stochastic Structure Model (SSM)

SSM is a simulated model, preferably based on analytical and/or heuristic rules for the tree growth; however, any viable algorithm for generating tree forms may be used. Importantly, the ultimate output of the SSM simulation is a table containing data sets *U*, describing the tree structure.

Additionally, SSM may be supplied with stochastic variability in its parameter values. Through our studies we implement simple stochastic variations in the form of normal and uniform distributions added to the parameter values of SSM.

Finally, the elementary units, such as cylinders [31], forming the SSM branches should be compatible with the units used in the QSM reconstruction.

Examples of SSM are: *LIGNUM* [13] – a functional-structural plant model based on the physiological principles of growth of pine trees, but also applicable to other tree forms [32]; *self-organizing tree model* [3] based on the heuristic principles of growth, capable of producing various tree shapes and used in computer graphics; *plastic trees* [4] procedural growth models used in computer graphics; *AMAP/GreenLab* (see e.g. [33, 34]) modeling approach to generate FSPM based upon empirical rules of growth with some physiological processes taken into account.

In this work, we use the self-organizing tree model (SOT) with shadow propagation algorithm [3] as SSM adapted for comparison with QSM. Note that more specialized tree growth models designed for the species in question would be better for the morphology optimization and the usual choice is FSPM's, see e.g. [30].

Structural data sets (U)

Structural data sets for any given tree structure are empirical collections of the physical dimensions and spatial orientation measures of segments and branches that are composed of segments. These data sets must be similarly obtained for any pair of $\{U_m,U_d\}$ in the calculation of the structural distance.

Quantities in the data sets may represent scalar characteristics and/or relations between several covariates (e.g. radii, lengths, angles, tapering function of a branch etc.). On one hand, one needs to exhaustively describe morphology of the tree using various geometrical and topological features. On the other hand, as the number of compared data sets $\{U_m,U_d\}$ grows the efficiency of the optimization

routine decreases, since the number of distance measures to be minimized grows correspondingly (one distance value for each pair $\{U_m,U_d\}$). In case of multiple data sets, we minimize the average value of the corresponding distances.

Branch- and segment-related data are described in Table 1 and Fig. 8 (in Methods). These features are not exhaustive and can be augmented when needed. But we found this set sufficient for obtaining realistic tree shapes. Throughout the manuscript we maintain the notations B^w and S^w for the branch- and segment-related data sets of the (Gravelius) order w, respectively. The zero order w is assigned to the trunk (a branch connecting a tree with the ground). At the branching points, the lateral buds give rise to branches with order $w+1$, where w is the order of the parent branch, while the apical buds continue the branch of the same order.

Table 1: Branch and segment features.

**Branch features, units**	Description
β, degree	Inclination angle of the branch, i.e. angle with its parent branch.
α, degree	Azimuthal angle of the branch, i.e. angle around its parent branch (calculated from the fixed direction).
L_t, m	Total length of the branch (calculated as the sum of the segment lengths constituting the branch).
R_f, m	Initial radius of the branch, i.e. radius of its first segment.
L_a, m	Length of over the parent branch from its beginning segment to the point where the current (child) branch emanates.
**Segment features, units**	Description

R, m	Radius of the segment.
L, m	Distance from the beginning of the branch to the segment.
γ, degree	Angle between horizontal projections of the segment and its parent.
ζ, degree	Angle between vertical projections of the segment and its parent.

The additional details of the data set representation are explained in Methods.

Measure of structural distance (D_S)

The distance D_S between any two data sets, or empirical distributions, measures the difference between the local densities of points in *U*-space for these data sets, i.e. *S* and *B* tables of morphological features. Here, it is constructed by measuring SSM vs. QSM difference of the normalized cumulative distributions of the point densities projected onto a number of line directions in the coordinate space of the variables in *U* (Fig. 9A, in Methods). The difference between the projected cumulative distributions is further measured by the Kolmogorov-Smirnov statistic. The resulting distance between the two data sets *U* is an average of all statistics calculated from each of the lines.

In order to provide a reference to traditional measurement systems, we also calculate three main tree characteristics that are used for describing a tree shape [35], that is *height* (*h*), *girth* (*g*), and *crown spread* (*c*). Finally, to compare SSM and QSM shapes we calculate relative error distances d_h, d_g, and d_c for height, girth, and crown spread, respectively. The classical distance d_i shows how large is the difference between entities *i* of the two trees in proportion of the corresponding reference/QSM tree value.

The details of the distance calculations are further explained in the Methods section.

Optimization routine

The measure of structural distance $D_S(U_m, U_d)$ is minimized by adjusting the parameters v of SSM. With infinite sampling, $D_S = 0$ for two trees that have exactly the same parameters v. These trees are not copies of each other, but they are structurally similar. The choice of U defined in D_S is not unique, but ideally U should satisfy the following uniqueness condition for D_S to yield an acceptable measure of distance. Let three trees be defined by the corresponding parameters v_A, v_B, and v_C with the data sets U_A, U_B, and U_C, respectively. Then, if $D_S(U_A,U_B) < D_S(U_A,U_C)$, one can update $C \leftarrow B$, i.e. substitute tree C with tree B, find any new v_B for which the inequality holds, and repeat until $D_S(U_A,U_B) \rightarrow 0$ and $v_B \rightarrow v_A$. In practice, this should be true in a large neighborhood of v_A; however in practice, $D_S > 0$ due to the finite sampling and insufficient model.

III. Testing of the algorithm

First, we run the optimization within each of the parameter groups $I - V$, representing different processes of growth (see Methods for details), to determine the basic values of the parameters. These basic values represent choices that generate a viable tree structure similar to the target QSM. Each optimization run takes the best parameters for the group optimized at the previous step. The target structural distributions U for these runs are segment-related (S) features of the branches of topological order w = 0, 1, i.e. $S^{0,1}$. Note that this exercise serves a basic exploration of the model's behavior, which can be (partially) replaced, for example, by the expert guesses for the parameter values or some calibration process.

Second, based on these preliminary results we may want to determine the most influential parameters for each of the group and combine them in a single optimization set up. Changes in these parameters cause the largest relative changes in the structural distance value. This step is required to reduce optimization time, and it is not needed if one possesses large enough computational resources. Several independent optimization runs were taken in order to determine the most influential parameters. For example, we found that the angular properties vary the least among these runs, whereas the apical dominance requires subtler adjustments (as can be understood from the complex structure of the target QSM).

Low order topological adjustment of the shape

After these initial manipulations, we obtained a model with 11 parameters and good fit of the trunk (w = 0) and first order branches (Fig. 3C) with classical metrics d_h = 0.05, d_g = 0.42, d_c = 0.57. However, the overall form of the resulting minimal score tree does not resemble the target QSM due to its rosette-shape (Fig. 3A, B). A closer look at the tree reveals that the higher order branches (w > 1) are mainly responsible for the formation of the rosette-shape of the tree, i.e. the orders which were not subject to the optimization (Fig. 3A, E). This example demonstrates the contribution of the higher order branches to the overall tree shape, which suggests using the information at w > 1 in further optimization steps. Moreover, the branch-related (B) features, such as the angular properties of branches of order w > 1, were not captured well (Fig. 3E), although similar order segment-related S-features show correct stochastic tendencies (Fig. 3D) generated automatically by the growth algorithm of the SSM. However, note that these features of w > 1 were not subject to optimization.

Figure 3: The rosette-shape SSM resulting from adjustment of the low order segment-related scatters. (A) The SSM tree; (B) the target QSM; (C) some segment related ($S^{0,1}$) scatters used in the

optimization; (D) higher order (w = 2) S-scatters (not used in optimization); (E) higher order (w = 2, 3) branch related B-scatters (not used in optimization). SSM/QSM scatters are shown in red/blue.

Low and high order topological adjustment

The increase in number of the structural feature tables is coupled with the increase in number of distinct distance values, that is, each pair of tables (QSM vs. SSM) produces a distance score to be optimized. Although the optimization of the mean distance value for all tables hinders the improvement for each table separately, the low order and high order branches need to be fitted to the corresponding branches of the target QSM. To reduce the number of distinct feature tables for the optimization we further utilize a set of merged data sets resulting in two joint segment- (S) and branch-related (B) tables for all topological orders.

Thus, we opted for $S^{0,1}$ and $B^{2,3,4}$ merged data sets in the next run of optimization to account for the higher order branch variability (Fig. 4, d_h = 0.08, d_g = 0.20, d_c = 0.68). We observe a significant improvement to the tree form due to the correct account of the angular properties of the higher order (w > 1) branches (Fig. 4E). The poor convergence of the branch linear dimensions (radii, lengths etc.) present in the branch-related tables might be due to the parameter choice of the model. Namely, the small proportion of branches with similar R_f values (Fig. 4E) is the result of the fixed segment length we selected as a compromise between realism and computational complexity. The QSM minimal segment length is close to zero, median is 0.06 m, whereas that of SSM is fixed to 0.2 m. We note the similar span of the curvature data points of SSM and QSM for w = 1, 2 (Fig. 4C and D). The w = 2 branch curvature was automatically generated by SSM as a result of the correct proportions of the w = 1 branches, which were obtained during the optimization. Additionally, due to the lack of the

orientation landmark in the feature data sets our best-fit SSM is fitted to the target QSM with accuracy of the rotation around Z-axis.

Figure 4: Low and high order adjustment of the stochastic feature tables. The best-fit SSM is obtained through optimization against $S^{0,1}$ and $B^{2,3,4}$ merged feature data sets. (A) The best-fit SSM tree, (B) the target QSM tree, (C) some projection scatters from S^1, (D) S^2 projection scatters, (E) B^2 and B^3 projection scatters.

Clonal nature of the best-fit SSM

Due to the highly discrete and stochastic nature of the tree growth, the structural distance hyper-surface in the space of the parameters is extremely variable (Fig. 5A). Hence, finding the global minima of such surface is not a trivial task. The classical smooth function optimizers are not suitable in this case, while stochastic discrete optimizers, like the genetic algorithm, seem to be more appropriate. Moreover, the hyper-surface itself is a stochastic entity changing every time the new sample of random numbers is used for a particular SSM growth realization. Therefore, any best-fit SSM is the best for a particular realization of this stochastic process and one needs to study variability of the tree shapes (Fig. 5B). We call these many realizations of the SSM growth *morphological tree clones*.

Figure 5: Stochastic structure distance profiles in the parameter space. (A) Three realizations of the distance hyper-surface projection along a dimensionless parameter λ of the SSM, controlling the apical dominance of a tree (the shown fragment of the projection with the step of 0.001 approximates 30% of the allowed variability of the parameter during optimization, which was [0.0, 0.65]). (B) Structural distance values (with $U = \{S^{0,1}, B^{2,3,4}\}$) for 100 randomly generated SSM trees for each value of a discrete SSM parameter, i.e. number of growth iterations (red line connects the median

points of the distance distributions for each parameter value; blue line shows the same median distance profile but for the distance with $U = S^{0,1}$, see (C)). (C) Same as in (B), but $U = S^{0,1}$ (blue line is the median profile; red line is from (B)). The SSM is the best-fit SSM obtained in the experimentation reported in Fig. 4; the black arrow indicates the parameter value of the best-fit SSM found in the experimentation.

The structural distance profile depends not only on the parameters of the SSM, but the choice of the structural data sets. For example, in Fig. 5B and C the median distance profile is depicted given $U = \{S^{0,1}, B^{2,3,4}\}$ (red line) and $U = S^{0,1}$ (blue line). In the given parameter range the latter seems to be more flattened and lifted compared to the former. The addition of the $B^{2,3,4}$ data set might be seen as a perturbation to the distance profile changing the landscape properties (like minima). In our simulations we maintain the global parameter boundaries, which allows for a search within the full available space. However, we sequentially improve the model characteristics by perturbing the system, i.e. changing the parameters, their intervals, and the U data sets to address problematic parts of the SSM (like rosette-shape, Fig. 3) such that at every next optimization run the genetic algorithm is instructed to search around the previous best point using the initial ranges (see the genetic algorithm in Methods).

Given the considerations above about the nature of the structural distance hyper-surface, the further study of the morphological clones is needed. Specifically, the variability and plausibility of the clonal shapes need to be addressed. For example, the clones must be further selected as to produce realistic tree shapes, however, in our analysis we did not find any unrealistic tree sampled from the best-fit SSM. Additionally, the variability of the clones can be further calibrated, for instance, by the analysis of the natural/QSM clonal individuals.

Morphological tree clones

The main objective of our work is the generation of the morphological clones. In our pipeline, this occupies the last stage (see Fig. 1, B5). After the optimization is finished and the best-fit SSM is found, one can further randomize the outcome of SSM by letting the random number generator produce different sequences every time SSM is run. As a result, the different realizations of SSM should constitute the morphological clone generator yielding structural copies close to QSM and to each other and *varying* in fine detail of organization of their branches. In other words, the coarse-grain structure is repeated in each clone (and possibly grasps that of the target QSM), whereas the fine-grain structure varies.

Figure 6: Morphological clones generated from the best-fit SSM. The best-fit SSM was found using the higher topological order adjustments (Fig. 4) with number of growth iterations 30 (A), 26 (B), and 18 (C). The height, girth, crown spread, and classical metrics distributions are shown in (D) for the clones in (A), (B), and (C) (the total number of generated clones for each case is n = 100, only 6 are shown). The black horizontal line indicates the corresponding measure of the target QSM.

We demonstrate visualization of six clones for three distinct models in Fig. 6 (clones from other best-fit SSM's are provided at [36]). One can see the fine-grain variation in the structure in each panel of the figure, although the overall (coarse-grain) structure is preserved and presumably captures that of the target maple QSM from Fig. 2 (however, the models have higher branch densities than the QSM, due to the discretization of the space using the voxels of finite size, see [3]). The three models are: the one found during the optimization process (Fig. 6A), the one minimizing the sample median distance profile for $D_S(U = \{S^{0,1}, B^{2,3,4}\})$ shown in Fig. 5B (Fig. 6B) and one minimizing the sample median profile $D_S(U = S^{0,1})$ from Fig. 5C (Fig. 6C).

Out of 100 simulated clones for each case, we can see that the best-fit SSM obtained directly as the optimization outcome (Fig. 6A) produces larger proportion of individual trees exhibiting the three standard allometric measures closer to those of QSM (Fig. 6D). However, we argue that such simple description of a tree, as using the allometric measures, cannot be exhaustive enough to capture both the overall structure and its fine details.

The height statistics have the largest discrepancy amongst the three models in Fig. 6 but by the visual inspection of the drawn clones one can see that this variability does not exert significant alterations of the Z axis span and the trees seem to have even heights. Perhaps, the way we calculate the height of a tree produces such large deviations in each particular case, which makes it a non-robust estimator for modeling quality. That is, the overall branch density may not be affected if a single branch protrudes higher than others, thus the overall outlook of a tree is not affected whereas its height is.

Note also that the model clones in Fig. 6A are the direct result of the optimization, i.e. the best-fit SSM. The clones in Fig. 6B and C are obtained by manually adjusting the growth parameter to minimize the median distance profiles from Fig. 5B and C, respectively. The growth iteration parameter directly affects the tree height. Hence, the larger proportion of clones with heights closer to the QSM height is achieved in case of the best-fit SSM (Fig. 6D). Additionally, this indicates that the optimization implicitly accounts for such simple allometric measure as height.

Similarly, the girth estimation, although being captured correctly, produces large errors d_g, which seems to be a result of variation in its linear dimensions (Fig. 6D). The girth dimension spans a small proportion of the dimension of the whole tree: from several to tens of centimeters compared to meters of the whole tree. This makes the girth specific error look gigantic (exceeding in some cases 100%) and thus non-robust as well.

The crown spread measure shows significant variation (Fig. 6D). We believe that this takes place due to the environment of the real tree the QSM was reconstructed from, which was not modeled appropriately in the SSM. Namely, the environmental effects (positions relative to the sun, as the tree grows in the Northern country, animals, winds, neighboring trees etc.) might cause systematic influences exerted on the shape of the QSM tree. These influences were not accounted for in the SSM, which was allowed to grow in any direction, limited by the uniform light conditions, existing branches of the same tree, and global boundaries of the available space. In addition to the environment influences, there are TLS measurement and QSM reconstruction errors, arising from the physical limitations of the instrumental technique and stochasticity of the QSM formation, respectively.

Finally, the true understanding of the variability of any measures of the morphological clones comes with the measurements of the real clones, i.e. trees similar in shape and/or genetically. Carrying out control experiments with QSM reconstructed from the real clonal individuals can only assess the variability. These real clone controlled experiments can further identify whether the obtained variability is large/small for the given species/clones and lead to the adjustment of the optimization parameters.

IV. Performance of the algorithm

We have performed several tests on the performance of the algorithm. The most computationally intensive parts of the algorithm are the SSM simulation and optimization, which both depend on a particular SSM implementation and the parameter search space.

Computational time and data size scale linearly, i.e. $O(N)$, with the number of morphological features when extracted from a tree growth model.

Next, we used surrogate data, namely standard normal distributions, for each of the features to assess the computational complexity of the distance algorithm. The results of this numerical assay are shown in Fig. 7.

Figure 7: Computational complexity of the distance algorithm. CPU time (*s*) vs. number of line projections (A), number of structural features (B), and number of samples per structural feature (C). Specification: a single 2.9 GHz core is used; where fixed, number of features is 20, number of samples is 1000, number of line projections is 1000 (B) and 500 (C).

V. Bayes Forest toolbox

We developed a unified interface using Matlab (MATLAB, RRID:SCR_001622) to facilitate exploration, drawing, optimization, and simulation of SSM and QSM as well as to study the morphological tree clones. Our interface allows for faster and easier manipulation of the required data, models, and optimization routines from the Matlab Optimization Toolbox, using only the required elements of otherwise complex Matlab configuration for the analysis.

The Bayes Forest toolbox is freely available at [36] (the version used in this study) and at [37] (the link is preferred for contributions and contains the latest version of the package). We also encourage the plant and computer scientists' community to expand their efforts using the toolbox with other species and models. Such a systematic approach can further be useful in tinkering the best options for creating QSM, SSM, and construction of the structural data sets.

VI. Discussion

In this work, we described an algorithmic pipeline aimed at producing stochastic structural replicas, or morphological “clones”, of trees from a QSM tree (based on TLS data) and a complimentary SSM tree. The pipeline is based on an iterative minimization of a distance between morphological structures. The distance is based on construction of the structural data sets of the tree morphologies and subsequent measure of their discrepancy using the ideas of distribution tomography analysis. The resulting best-fit morphological clones are statistically similar which is expressed in overall similarity of their form, but difference in fine details of structural organization.

Here, we have shown the general logic behind the pipeline for generation of the morphological clones. For this purpose we used a highly variable procedural tree model [3], which is more difficult to optimize. As the pipeline consists of several elementary steps, each of which can be changed according to the application and target analysis, we have proposed an initial set-up and basic configuration. We assume larger possibilities of exploration of the proposed configuration, let alone changing the steps and individual algorithms within the pipeline, which could be fulfilled by the community of plant science researchers.

The interest of building this pipeline was driven for biological applications rather than visualization purposes. Thus, for example, we use real TLS measurements and general-purpose measure of the distance, while omitting visual effects (e.g. shades, leaves etc.). We believe this pipeline can be useful in the rigorous analysis of the plant morphogenesis and corresponding applications, which differ from similar studies done in computer graphics field, e.g. [5].

Moreover, our algorithm makes use of the distance measure taking into account significant portion of the data for at least one topological order. This allows for a more comprehensive analysis of forms and their description, using empirical distributions of morphological features rather than scalar allometric entities. Due to this reason, we do not rely on the traditional metrics comparison in this work as we found that similar values for the height, girth, and crown distances may correspond to different tree forms and, thus, be non-robust.

Use of several QSM trees can enhance the robustness of the statistical analysis presented here. In this case, similarly looking trees should be used and the degree of similarity might be established using our definition of the structural distance. For example, the trunk features are more reliably reproduced in statistical sense, when several QSM's are used. It might be stressed that other notions of "clone" can be used to establish relationship with morphology. Thus, the genetic clones might be utilized to establish to what degree the morphology of a tree is encoded into genes.

Methods

Laser scanning measurements

The subject tree was measured in leaf-off conditions and our system consisted of a phase-shift based terrestrial laser scanner, namely the Leica HDS6100 with a 650–690 nm wavelength. The distance measurement accuracy and the point separation angle of the scanner were about 2–3 mm and 0.036 degrees, respectively. The horizontal distance of the scanner to the trunk was about 7–12 m, giving an average point density on the surface of the trunk (at the level of the scanner) for a single scan around 2–5 points per square centimeter. The QSM of the subject maple tree consists of 19,000 cylinders approximating 3,078 branches.

Self-organizing tree model (SOT)

In this work we used SOT implemented in the LPFG simulator, part of the Virtual Laboratory software suite [38], version 4.4.0-2424 for 64-bit Mac OS, see [39]. This procedural tree model is fast and able to generate variety of forms.

The total number of growth parameters of the model is 27: 23 are grouped, 4 are fixed. The values of the latter are dictated both by suggestions of the authors in [3] and the compromise between computation time and details of the morphological description. For example, the segment length is 0.2 m, the voxel size is 0.2 m, and the model tree grows within 12x12x12 m cube from the center of XY bottom plane of the cube (Z-axis is oriented upwards).

The grouped parameters are divided between 5 distinct groups corresponding to different related processes:

Group I: the initial growth parameters, including limiting values, and pipe model related parameters.

Group II: environmental effects such as sensitivity to the neighborhood shading, vertical gradient distribution of the light, tropism etc.

Group III: apical dominance parameters.

Group IV: shadow propagation related constants (see [3]).

Group V: angular/branching properties.

Data set representation

One needs a more compact representation of the data, since the larger number of data sets U is, the larger number of distance values is and the more difficult the optimization becomes. One solution is to use larger data sets with all application specific features. Therefore, we use tables including all measured features; hence, one table represents a data set. However, it is not possible to merge segment- and branch-related features into a single table as these differ in dimension (usually one branch is composed of many segments). Thus, we usually compare the array of pairs $\{U_m,U_d\}$, having as a result the array of distance values, but with such larger table representation we have smaller size of these arrays. For example, one could form U_1 from inclination angle β and U_2 from azimuthal angle α, or, alternatively, from a larger table U consisting of all branching features (β, α, L_t, R_f, L_a from Table 1). The former distance would be formed from D_1 and D_2 for U_1 and U_2 respectively, whereas the latter case would require a single distance to optimize.

Figure 8: Visual structure of a tree and its representation using the structural data sets *U*. (A) A sample tree; (B) geometrical features of the branch- (*B*) and segment-related (*S*) data sets; and (C) various projections of the *U* data sets.

Additionally, it is possible to merge the corresponding data sets for several topological orders, which results at most in two large data sets of branching and segment features, respectively. While this simplifies the search of the distance minimum, this technique must be used with care as in this case one heavily relies upon the growth rules of SSM. If these rules are not based on biologically motivated rules, SSM can produce highly unrealistic tree forms as the “best-fit”, since there is a possibility to mix the features of different topological orders. For example, the branches of higher order could be much thicker than those of the lower order, which should not happen using biologically based growth algorithms (e.g. pipe model).

In a simulated SSM structure the extraction of topological relations between branches is straightforward: the lateral buds start the next order and apical buds continue the current order. However, this is not the case with QSM since it is a time snapshot of a tree form that does not retain the history of the tree growth. Thus, the reconstruction algorithm requires other means for extracting the topology. Although the reconstruction algorithm defines a complicated procedure that outlines the topology of a tree, it can be roughly approximated by the following rule: at branching points the thickest branch is the continuation of the same order w, while thinner branches are lateral expansions of the order $w + 1$ [18]. For the species with weak apical dominance (shrubby trees) we follow the rule when extracting topology from SSM (for the species with strong apical dominance, the rule gives the same result as the analytical procedure).

Structural (D_S) and traditional distances

The structural distance is calculated measuring difference between the normalized cumulative distributions of two point densities projected onto a number of line directions. The directions of lines are generated with quasi-Monte Carlo method using low-discrepancy (quasi-random) sequences, which cover the given space more evenly than uniformly generated sequences.

The empirical probability density function $p(U)$, $U \in R^N$, can be approximated by the series of 1D density functions $p_{1D}(U,L)$, where L is a line in R^N. Each of these 1D functions is constructed by projecting all the data points of U onto a line L (in this work we used 1000 lines). Cumulative distributions $P_{1D}(U_m,L_i)$ and $P_{1D}(U_d,L_i)$ (U_m and U_d being the two point densities, e.g. SSM vs. QSM data sets) for each line direction L_i are compared, and for any given data set pair $\{U_m, U_d\}$ the resultant distance value is:

$$D_S(U_m, U_d) = \frac{1}{n}\sum_{i=1}^{n} K[P_{1D}(U_m, L_i), P_{1D}(U_d, L_i)],$$

where n is the number of lines and operator $K[\cdot,\cdot]$ returns the Kolmogorov-Smirnov statistic for the given pair of 1D empirical cumulative distributions.

Figure 9: Distribution tomography of the structural data sets (A) and classical metric for the crown spread (B). (A) Data points in U (projected here for simplicity onto (u_i,u_j) plane, i.e. in 2D) are used to construct the projection onto a line L. Cumulative empirical distribution is calculated along L (red). Only one line is shown. (B) Top view of a tree: spokes (red) emanate from the ground segment (green) extending up to the most distant points (blue).

Height (h) is calculated as the highest point of a tree. *Girth* (g) is calculated as the diameter of the ground segment, because the diameter at breast height is not appropriate for the shrubby trees. *Crown spread* (c) is calculated as follows. First, on XY-plane (top view, Fig. 9B) the set of spokes (red lines in Fig. 9B) emanating from the center of a tree (the ground segment, green circle) is built with 10 degrees azimuthal separation. Then the length of each spoke is calculated as a distance from the tree center to the most distant point of the crown in the direction of the spoke (blue circles). The crown spread is twice the average of all spokes of a tree.

Finally, when comparing two tree shapes with traditional metrics we calculate the distances as follows:

$$d_h = \frac{|h_d - h_m|}{h_d}; d_g = \frac{|g_d - g_m|}{g_d}; d_c = \frac{|c_d - c_m|}{c_d}.$$

In this, h_d, g_d, and c_d are the height, girth, and crown spread of the QSM tree, respectively, whereas h_m, g_m, and c_m are the corresponding attributes of the best-fit SSM tree.

Genetic algorithm

Any algorithm from a standard optimization library (e.g. Matlab Optimization Toolbox) that finds a minimum of an objective function ($D_S = F(v)$) can be used. However, to facilitate global minimum search and given the nature of the problem we use the genetic algorithm (implemented in Matlab, version R2015b). Additionally, some parameters of SSM may take only integer values, so the genetic algorithm handles the integer parameters correctly unlike, for example, the classical steepest decent algorithm. The genetic algorithm iteratively finds a minimum of D_S, each iteration being called *generation*. Each generation is characterized with a number of individuals, i.e. *population*; one individual is equivalent to one set of the parameter values. The variation is controlled by the *crossover rate* (rate of recombination of the population parameters) and *mutation rate* (rate of introduction of the new variability into the population). The former is fixed to 80% in the Matlab Optimization Toolbox, whereas the latter is controlled by our configuration (19% in the rosette-shape example, Fig. 3, and 15% in the best-fit SSM from Fig. 4). The user controls values' ranges of the parameters. There are two types of ranges: *global* lower and upper boundaries for each of the parameter values and *initial range*, from which the algorithm tries to construct the initial population. The latter impacts the convergence rate: if it is too broad poor convergence is attained. Finally, the algorithm stops when it reaches a fixed number of generations without improving the distance.

Thus, the objective function takes the input parameters v, simulates SSM with v, calculates and returns structural data sets U_m. Subsequently, the objective function calculates $D_S(U_m, U_d)$ and returns it to the optimization routing. The SSM, being a stochastic model, *must* have a fixed random generator seed during optimization, i.e. the same input parameter set must produce the same structural output. This is needed for convergence of the optimization. After obtaining the final best-fit form of SSM, one can

further explore the variability coming from different random number sequences used in the SSM simulations. Such random best-fit SSM is capable of producing the clonal morphologies.

Availability of supporting source code and requirements

Project name: BayesForest

Project home page: https://github.com/inuritdino/BayesForest/wiki

Operating system: Platform independent

Programming language: Matlab

Other requirements: VLAB software suite, version >= 4.4.0-2424

License: MIT

Data availability

All data needed to reproduce the results of this study, some additional materials, and Bayes Forest Toolbox are available at [36]. The most recent version of the Toolbox is also available at [37] (this interface is preferred for the contributors and also contains the most recent version of the software). Tutorials on how to run the tests using the Matlab toolbox are available in the BayesForest Wiki [40] and snapshots are also available in the GigaScience repository, GigaDB [41].

List of Abbreviations

FSPM – functional-structural plant model.

QSM – quantitative structure model.

SSM – stochastic structure model.

SOT – self-organizing tree model.

TLS – terrestrial laser scanning.

Ethics approval and consent to participate

Not applicable

Consent for publication

Not applicable

Competing interests

The authors declare that they have no competing interests

Funding

This work was supported by the Academy of Finland: Suomen Akatemia (Center of Excellence in Inverse Problem Research, one of the PI's is Mikko Kaasalainen).

Author contributions

IP performed all simulations, processed the data, and wrote the manuscript; MJ wrote the code for calculating the structural distance, discussed the results; MÅ contributed to Bayes Forest Toolbox; PR generated and provided for the QSM data, wrote the manuscript and discussed the results; MK conceived the study, discussed the results, and wrote the manuscript.

Acknowledgments

We would like to thank Risto Sievänen and Wojtek Palubicki for useful discussion and comments on the model design and implementation.

References

[1] Prusinkiewicz P. Modeling plant growth and development. Current Opinion in Plant Biology. 2004;7:79-83.

[2] Fourcaud T, Zhang X, Stokes A, Lambers H, Körner C. Plant Growth Modelling and Applications: The Increasing Importance of Plant Architecture in Growth Models. Ann Bot. 2008;101:1053-1063.

[3] Palubicki W, Horel K, Longay S, Runions A, Lane B, Mech R, et al. Self-organizing tree models for image synthesis. ACM Transactions on Graphics. 2009;28:58.

[4] Pirk S, Stava O, Kratt J, Abdul Massih Said M, Neubert B, Mech R, et al. Plastic Trees: Interactive Self-Adapting Botanical Tree Models. ACM Transactions on Graphics. 2012;31:50.

[5] Stava O, Pirk S, Kratt J, Chen B, Mech R, Deussen O, et al. Inverse Procedural Modelling of Trees. Computer Graphics Forum. 2014;33:118-131.

[6] Hallé F, Oldeman R, Tomlinson P. Tropical trees and forests: An architectural analysis. Berlin: Springer; 1978.

[7] Sachs T, Novoplansky A. Tree from: architectural models do not suffice. Israel J Plant Sci. 1995;43:203–212.

[8] Room P, Hanan J, Prusinkiewicz P. Virtual plants: new perspectives for ecologists, pathologists and agricultural scientists. Trends Plant Sci. 1996;1:33-38.

[9] Sievänen R, Nikinmaa E, Nygren P, Ozier-Lafontaine H, Perttunen J, Hakula H. Components of functional–structural tree models. Ann Sci. 2000;57:399-412.

[10] Godin C, Hanan J, Kurth W, Lacointe A, Takenaka A, Prusinkiewicz P, et al., editors. Proceedings of the 4th International Workshop on Functional–Structural Plant Models, June 7-11, Montpellier, France; 2004.

[11] Mäkelä A, Hari P. Stand growth model based on carbon uptake and allocation in individual trees. Ecol Model. 1986;33:204-229.

[12] Rauscher H, Isebrands J, Host G, Dickson R, Dickmann D, Crow T, et al. ECOPHYS: An ecophysiological growth process model for juvenile poplar. Tree Physiol. 1990;7:255-281.

[13] Perttunen J, Sievänen R, Nikinmaa E, Salminen H, Saarenmaa H, Väkevä J. LIGNUM: a tree model based on simple structural units. Ann Bot. 1996;77:87-98.

[14] Lacointe A. Carbon allocation among tree organs: a review of basic processes and representation in functional–structural tree models. Ann For Sci. 2000;57:521-533.

[15] Rosell J, Llorens J, Sanz R, Arnó J, Ribes-Dasi M, Masip J, et al. Obtaining the three-dimensional structure of tree orchards from remote 2D terrestrial LIDAR scanning. Agric and For Meteor. 2009;149:1505-1515.

[16] Van Leeuwen M, Nieuwenhuis M. Retrieval of forest structural parameters using lidar remote sensing. Eur J For Res. 2010;129:749–770.

[17] Rutzinger M, Pratihast A, Oude Elberink S, Vosselman G. Detection and modelling of 3D trees from mobile laser scanning data. In: International Archives of Photogrammetry, Remote Sensing and Spatial Information Sciences. 2010;XXXVIII:520-525.

[18] Raumonen P, Kaasalainen M, Åkerblom M, Kaasalainen S, Kaartinen H, Vastaranta M, et al. Fast Automatic Precision Tree Models from Terrestrial Laser Scanner Data. Remote Sensing. 2013;5:491-520.

[19] Calders K, Newnham G, Burt A, Murphy S, Raumonen P, Herold M, et al. Nondestructive estimates of above-ground biomass using terrestrial laser scanning. Methods in Ecol Evol. 2015;6:198-208.

[20] Xu H, Gossett N, Chen B. Knowledge and Heuristic Based Modeling of Laser-Scanned Trees. ACM Transactions on Graphics. 2007;26:19.

[21] Livny Y, Yan F, Olson M, Chen B, Zhang H, El-Sana J. Automatic Reconstruction of Tree Skeletal Structures from Point Clouds. ACM Transactions on Graphics. 2010;29:151.

[22] Preuksakarn C, Boudon F, Ferraro P, Durand JB, Nikinmaa E, Godin C. Reconstructing Plant Architecture from 3D Laser scanner data. In: DeJong T., Da Silva D, editors. Proceedings of the 6th International Workshop on Functional-Structural Plant Models. 2010. 14-16.

[23] Hackenberg J, Spiecker H, Calders K, Disney M, Raumonen P. SimpleTree - an efficient open source tool to build tree models from TLS clouds. Forests. 2015;6:4245-4294.

[24] Kaasalainen S, Krooks A, Liski J, Raumonen P, Kaartinen H, Kaasalainen M, et al. Change Detection of Tree Biomass with Terrestrial Laser Scanning and Quantitative Structure Modeling. Remote Sensing. 2014;6:3906-3922.

[25] Raumonen P, Casella E, Calders K, Murphy S, Åkerblom M, Kaasalainen M. Massive-scale Tree Modelling from TLS Data. ISPRS Annals of the Photogrammetry, Remote Sensing and Spatial Information Sciences. 2015;II-3/W4:189-196.

[26] Smith A, Astrup R, Raumonen P, Liski J, Krooks A, Kaasalainen S, et al. Tree Root system characterization and volume estimation by terrestrial laser scanning. Forests. 2014;5:3274-3294.

[27] Kaasalainen M. Dynamical Tomography of Gravitationally Bound Systems. Inverse Problems and Imaging. 2008;2:527–546.

[28] Bracewell R. Numerical Transforms. Science. 1990;248:697-704.

[29] Sievänen R, Perttunen J, Nikinmaa E, Kaitaniemi P. Toward extension of a single tree functional-structural model of Scots pine to stand level: effect of the canopy of randomly distributed, identical trees on development of tree structure. Functional Plant Biology. 2008;:964–975.

[30] Potapov I, Järvenpää M, Åkerblom M, Raumonen P, Kaasalainen M. Data-based stochastic modeling of tree growth and structure formation. Silva Fennica. 2016;50:1413.

[31] Åkerblom M, Raumonen P, Kaasalainen M, Casella E. Analysis of Geometric Primitives in Quantitative Structure Models of Tree Stems. Remote Sensing. 2015;7:4581-4603.

[32] Lu M, Nygren P, Perttunen J, Pallardy S, Larsen D. Application of the functional-structural tree model LIGNUM to growth simulation of short-rotation eastern cottonwood. Silva Fennica. 2011;45:431–474.

[33] De Reffye P, Fourcaud T, Blaise F, Barthelemy D, Houllier F. A functional model of tree growth and tree architecture. Silva Fennica. 1997;31:297-311.

[34] Yan H, Kang M, de Reffye P, Dingkuhn M. A Dynamic, Architectural Plant Model Simulating Resource-dependent Growth. Annals of Botany. 2004;93:591-602.

[35] Frank E. A Numerical Method of Plotting Tree Shapes. Bull East Nat Tree Soc. 2010;6:2-8.

[36] Potapov I, Järvenpää M, Åkerblom M, Raumonen P, Kaasalainen M. Bayes Forest Toolbox. http://math.tut.fi/inversegroup/app/bayesforest/v1/. Accessed 25 June 2017.

[37] Potapov I, Järvenpää M, Åkerblom M, Raumonen P, Kaasalainen M. Bayes Forest Toolbox: the developmental version. https://github.com/inuritdino/BayesForest.

[38] Federl P, Prusinkiewicz P. Virtual Laboratory: an interactive software environment for computer graphics. In Proceedings of Computer Graphics International. 1999, p. 93-100.

[39] Prusinkiewicz P. Virtual Laboratory (VLAB) / L-studio homepage. http://algorithmicbotany.org/virtual_laboratory/. Accessed 17 Apr 2017.

[40] The BayesForest Wiki. https://github.com/inuritdino/BayesForest/wiki. Accessed 24 June 2017.

[41] Potapov I, Järvenpää M, Åkerblom M, Raumonen P, Kaasalainen M: Supporting data for "Bayes Forest: a data-intensive generator of morphological tree clones" GigaScience Database. 2017. http://dx.doi.org/10.5524/100337

Figure 1

Click here to download Figure Figure1.pdf

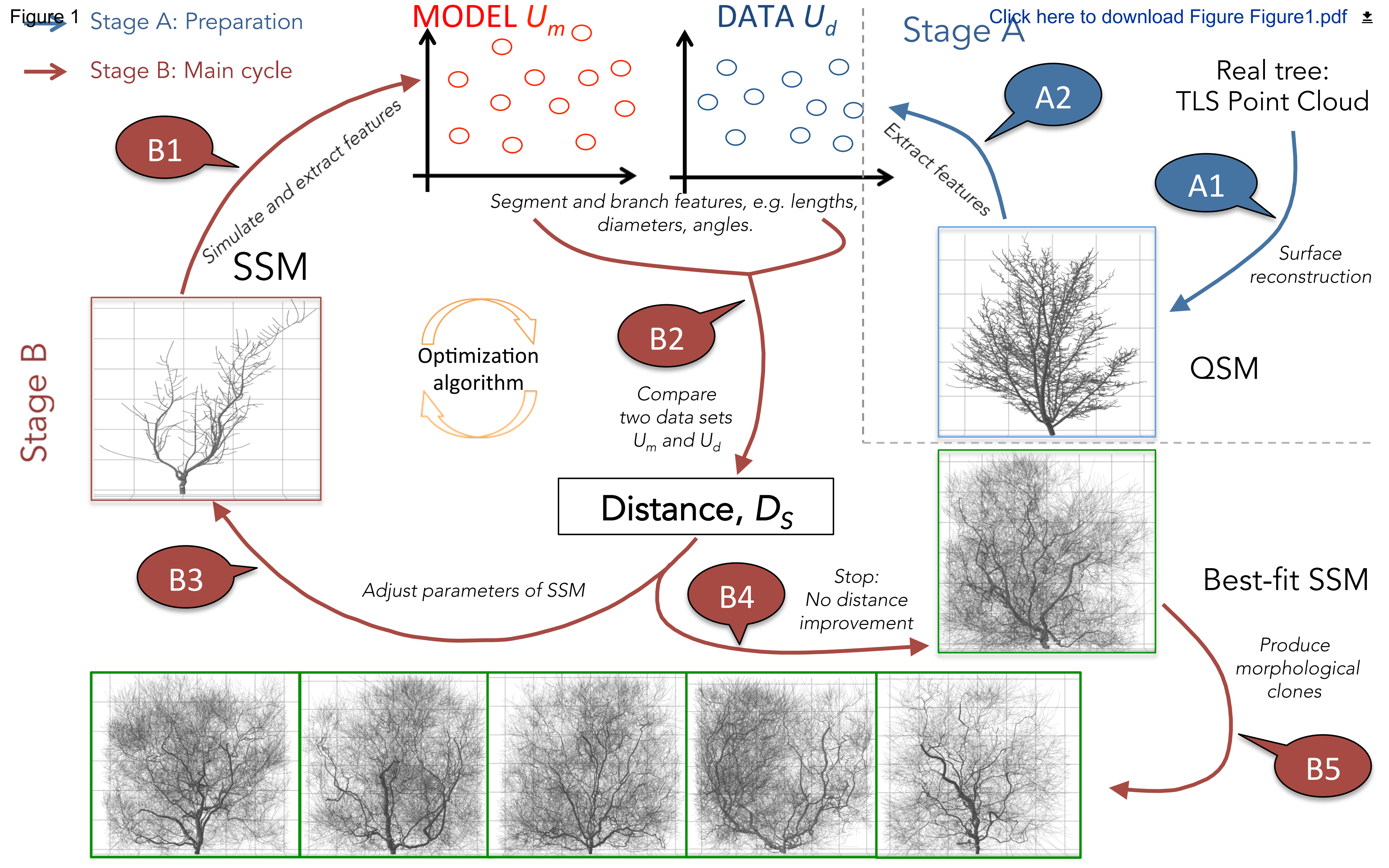

Figure 2

Click here to download Figure Figure2.pdf

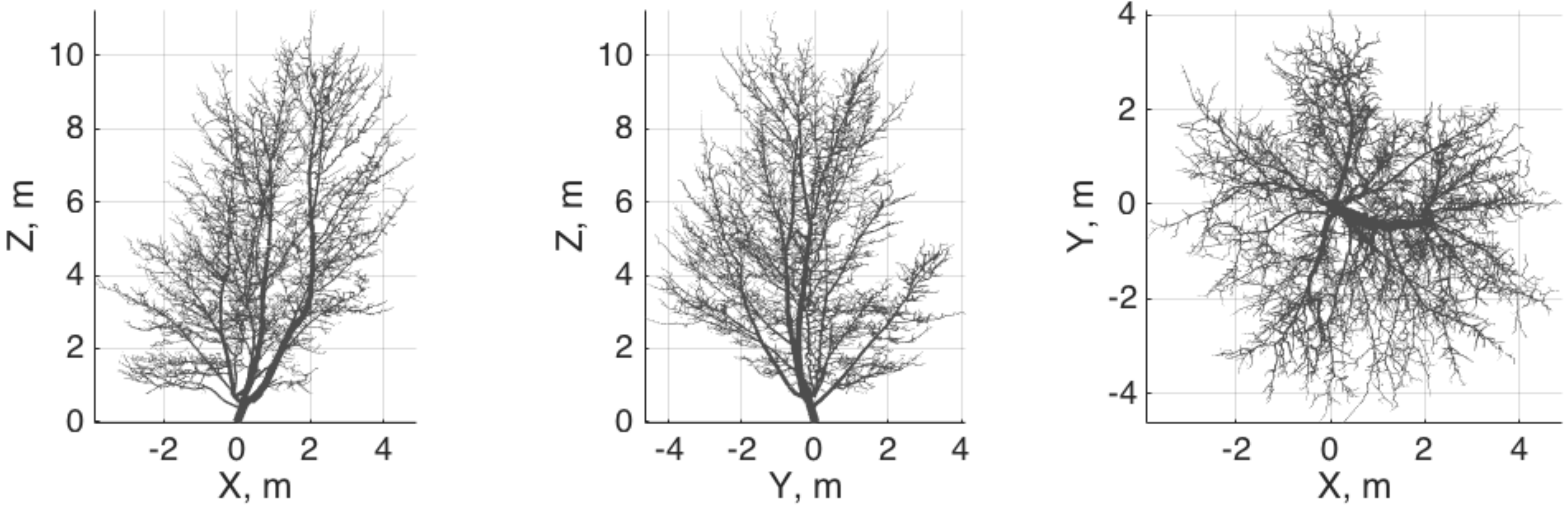

Figure 3

Click here to download Figure Figure3.pdf

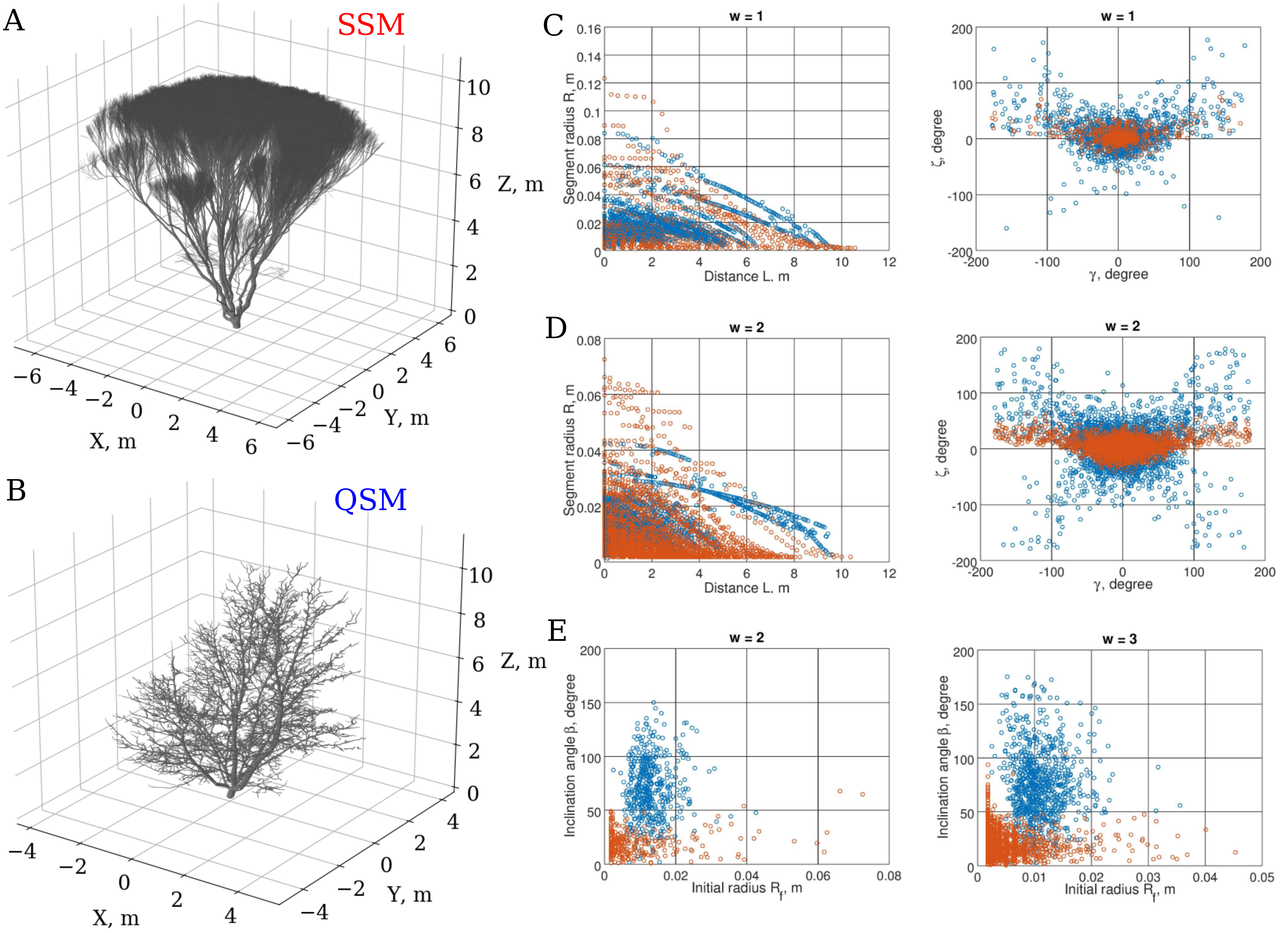

Click here to download Figure Figure4.pdf

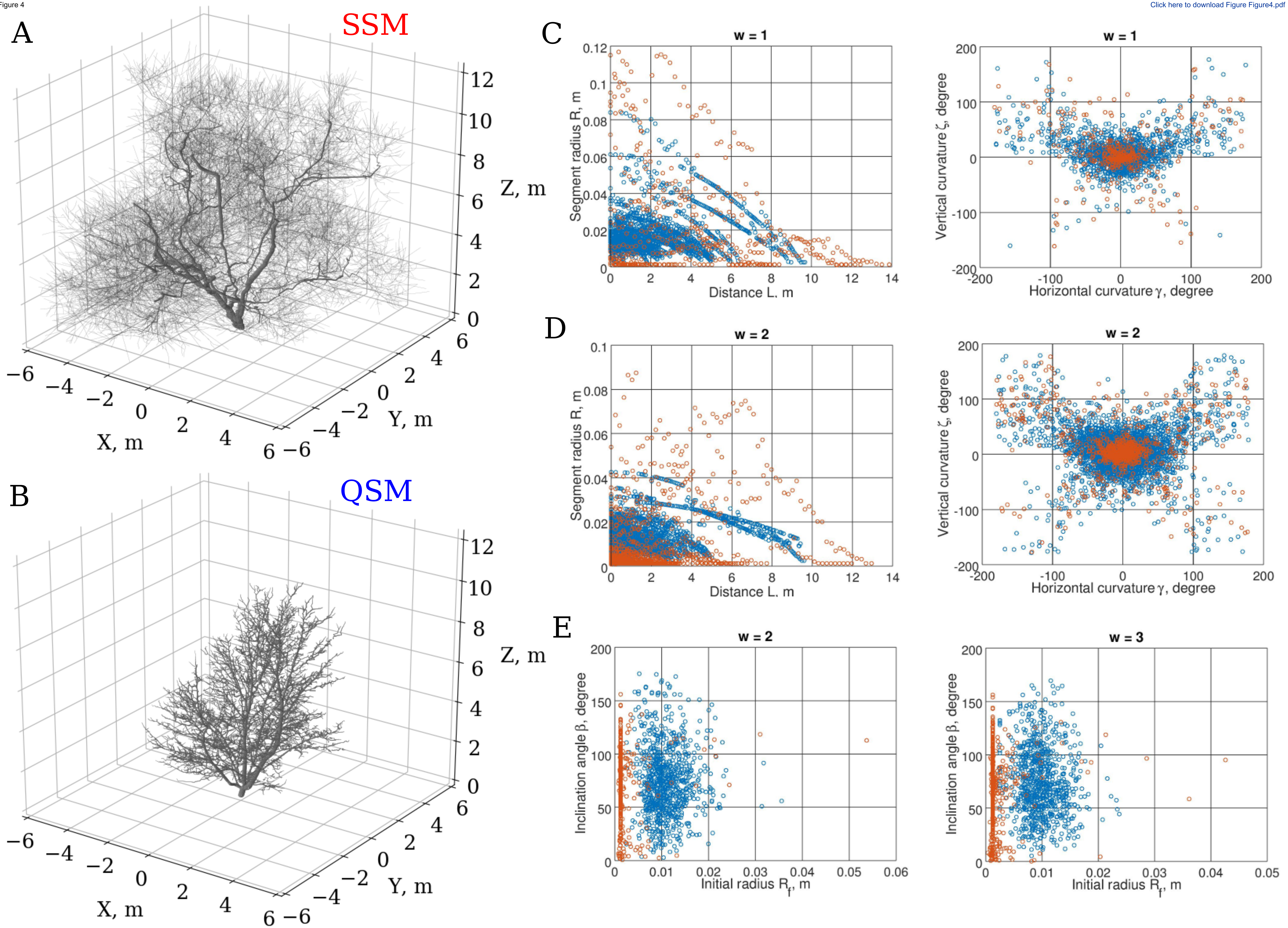

Figure 5

Click here to download Figure Figure5.pdf

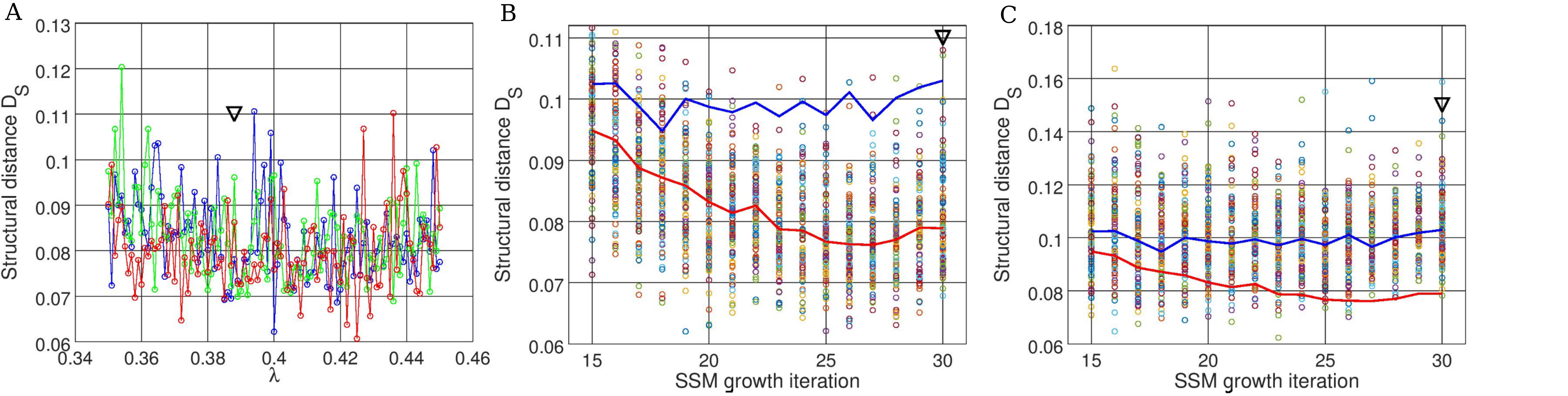

Figure 6

Click here to download Figure Figure6.pdf

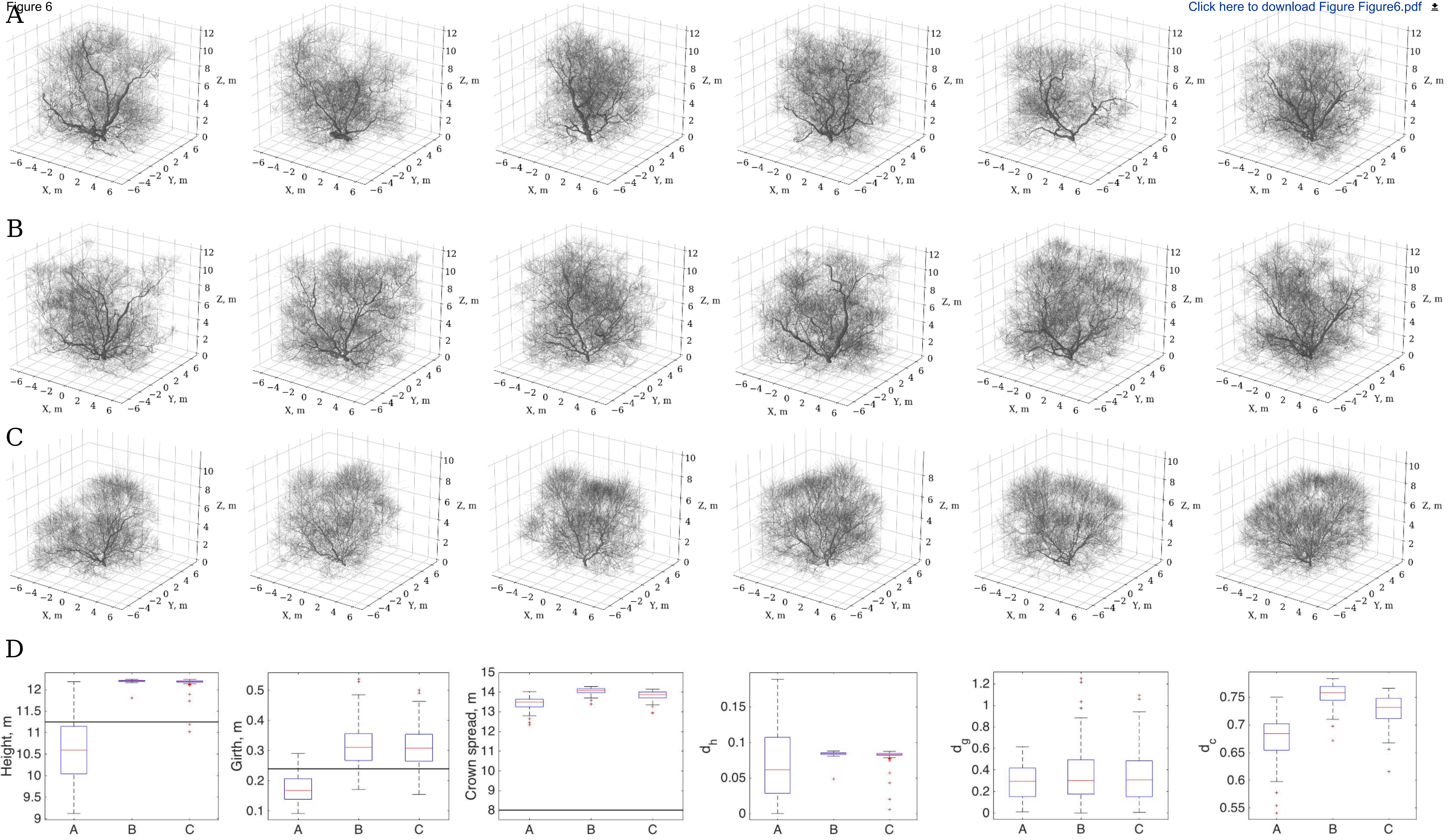

Figure 7

Click here to download Figure Figure7.pdf

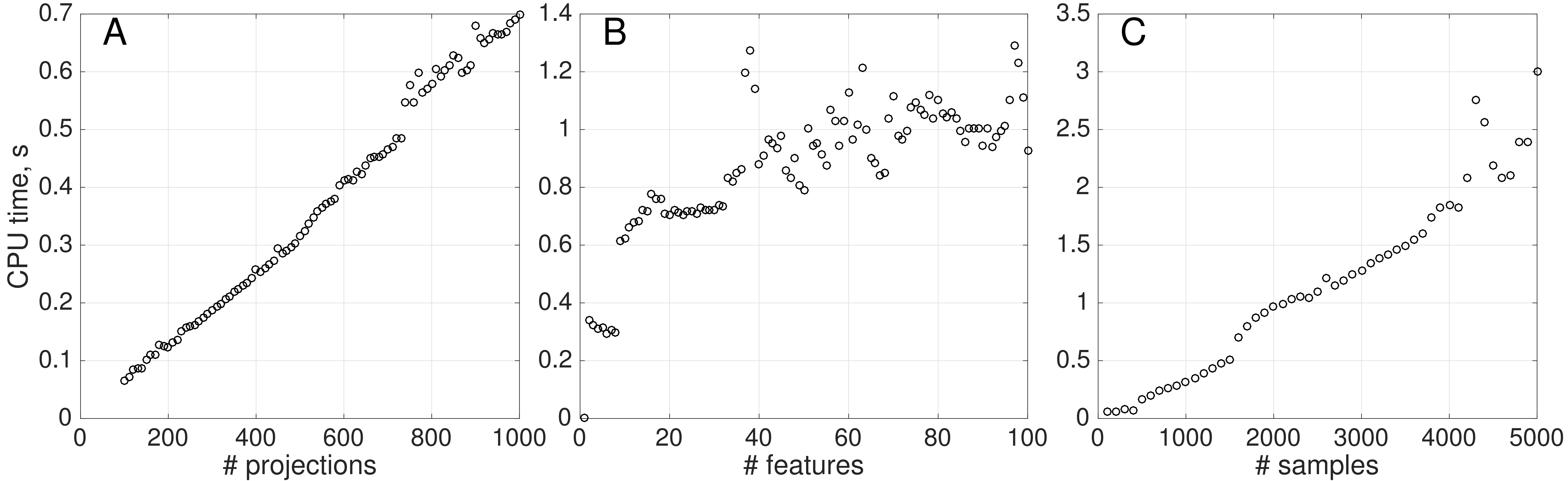

Figure 8

Click here to download Figure Figure8.pdf

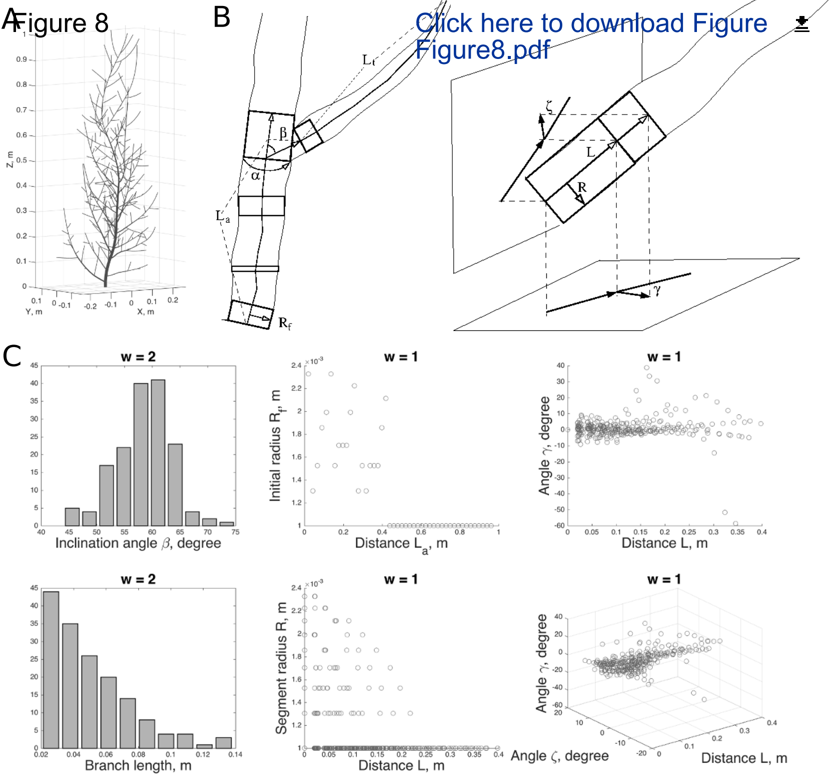

Figure 9

Click here to download Figure Figure9.pdf

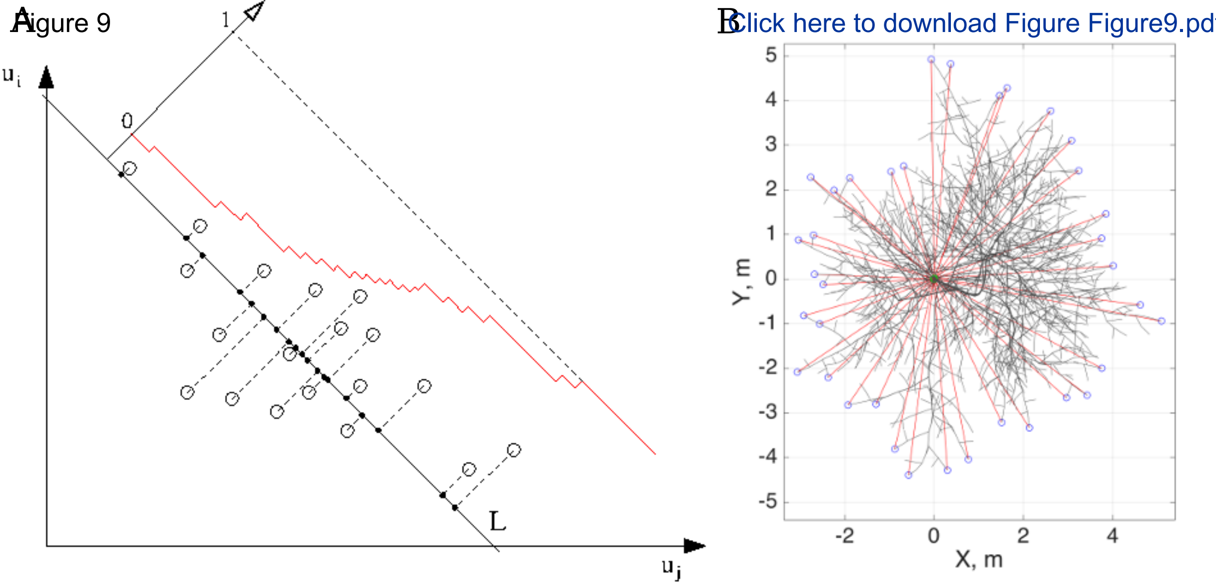

Supplement: GIGA-D-17-00087_Revision-1.pdf [file gix079_GIGA-D-17-00087_Revision-1.pdf]
