# Supplementary material for: Bayes Forest: a data-intensive generator of morphological tree clones
Source: Gigascience. 2017 Aug 19;6(10):1–13. doi: 10.1093/gigascience/gix079 (PMC5632294; doi:10.1093/gigascience/gix079)
Supplement: Response-to-Reviewer-Comments_Original-Submission.pdf [file gix079_Response-to-Reviewer-Comments_Original-Submission.pdf]

Author's Response To Reviewer Comments

We would like to thank the Reviewer #1 for his careful reading of the manuscript. Below we have addressed his concerns.

Reviewer #1:

1)"However, I found the section ordering a bit confusing. The methods section is found at the end of the manuscript, but many variables and concepts that are defined in this section are referred in the previous ones. This makes it difficult for the reader to follow the ideas and concepts explained without having to read ahead in the text. Depending on the editorial choice in the section ordering, moving the "methods" before "III. Testing for the algorithm" would certainly improve the reader experience. "

- We have revised the section ordering. We kept all new concepts we introduce as well as the notations before the main section "III. Testing of the algorithm". All additional technical and mathematical details were put in Methods as the journal's guidelines for authors suggest.

2) Another element which needs to be looked at is the extensive use of sub-sentence parts enclosed by parenthesis. It is generally not a good practice and breaks the text flow. Those should be either written as independent sentences or simply removed.

- We have revised this aspect of the manuscript.

3) Lastly, the manuscript has some grammar and language issues, which need to be addressed. Specific comments and recommendations can be found in the annotated PDF attached with this review report.

- We have closely followed the recommendations of the Reviewer. We are grateful for the thorough work he has done to improve the language of our manuscript.

We are thankful to the Reviewer #2 for the useful comments provided, specifically those regarding the performance of the algorithm.

Reviewer #2:

1) Ignoring the improvements in algorithm, how useful is the result of their Bayes-Forest (BF) from a plant science perspective? Have they been able to accurately emulate any plant characteristics that have resulted in better scientific understanding of plants.... or made things easier for plant phenotyping etc?

- This is a first attempt to generalize the Bayes-Forest concept into a unified interface. So far, we have not applied it to any particular real world situation. Our plan is to use the concept in various applications to test and refine the hypotheses (e.g. topology description in terms of Gravelius

orders may not be the best for shrubby trees) and, as a consequence, to improve the biological models. For example, our next project is to test correspondence between genetic clones and morphological “clones”, to separate the effects of environment and inheritance.

2) Can the authors take an example tree and compare their outcome with the FSPM of the same? Can the BF accommodate external abiotic factors? If not what are the limits of its usefulness.

- FSPM can be easily adopted into our approach as SSM. The goal of our approach is to make a statistically reliable comparison between the model and the real tree that is approximated with QSM. Any sort of FSPM can be used. Additionally, we have limited ourselves for certain choices of SSM, QSM, distance function, morphological features etc. These can be changed for the application specific purposes. For example, if FSPM is affected by the external abiotic factors, the parameters related to these factors can be certainly optimized against the target QSM structure. In fact, we have used parameters of the kind in the SOT model of this work and in another model (FSPM) of our previous study (Potapov et al., Silva Fennica, 50, 1: 1413, 2016).

3) Fig.1: Rework Fig. 1 to make the flow clearer. How is the empirical distribution Um obtained from the SSM? Give quantitative examples of stopping criteria for optimization. How are these thresholds chosen?

- We have remade Fig. 1 to improve the readability. Namely, we added: a color scheme to emphasize the data flow, small notes briefly describing each transition in the graph, a separation between the preparation stage and the main cycle.

4) Line 177: how the influential parameters quantitatively determined?

- We call insignificant such parameters that do not exert significant influence on the shape at least in the values' ranges studied. This group can be easily found by repeated runs of optimization with the same set up. These runs must land on a wide range of parameter values for the insignificant group. In other words, the structural distance hyper-surface is flat along these parameters. The rest of the parameters must belong to the significant group (besides, some technical parameters and those having no connection with shape of a tree).

To further clarify the term we have introduced the following statement: "Changes in these [influential] parameters cause the largest relative changes in the structural distance value."

5) Line 205 - 208: How are the relevant number of structural features determined? What is the scaling time/complexity and data size associated with these choices? provide a graph showing the relationship for a given plant type to give a better understanding.

- The number of relevant features is determined empirically; there are no analytical criteria to determine it quantitatively. Moreover, there is no analytical means to determine whether the given set of features is the best, complete, for a particular tree. Practically, features must account for both segment attributes (such as curvature in space) and branch attributes (such as length and angles). This gives the best optimization outcome. For example, one can always use our set of features as these were developed for a highly irregular tree shape (regularity of the shape, in

principle, must decrease number of relevant features).

When extracting features from SSM, adding branch and segment features scales linearly, i.e. as O(N).

Furthermore, we have empirically assessed the complexity of the distance algorithm with regards to number of structural features, sample size per feature, and number of line projections. The new figure was added to the manuscript.

Finally, we have added a new section "IV. Performance of the algorithm" discussing the computational issues.

6) Fig. 5 shows significant difference from target QSM values for several features. Only height seems to get close to the target. How useful are these results? Also what is the simulation time and computing requirements to achieve these results?

- The best-fit SSM derived clones (the A panel of the figure) show deviation only for the crown spread. This is due to the absence of external factors affecting the tree growth in SSM, for example, we do not model the effect of neighboring trees competing for the light resources, other objects (stones, hills) in the vicinity of the target tree, animals reshaping the tree etc. These factors contribute to the ragged XY projection of the crown of QSM. As we calculate the crown spread with high angular resolution (10 degrees) the total deviation of SSM from QSM can be significant.

Note also that the target QSM value (height, girth or crown spread), used as a reference, should not be close to the center (mean or median) of the distribution for the model clones. We do not know how often the QSM form is present among the clones. In other words, we do not know the variability of real clones. Thus, the QSM value might lie anywhere in the available distribution span, be it even an outlier.

The computational time is dependent upon a particular implementation of the SSM simulator. We found native Matlab codes to be extremely slow and suggest using compilable programs instead (like LPFG used in this study).

7) Provide a graph showing the scaling of optimization time/ efficiency as a function of the number of {Um,Ud} data sets.

- Using our distance measure it is impossible to assess optimization time/efficiency in a real set-up, as there is no way to compare any two distance values for arbitrary configurations. Also, we believe it is outside the scope of the paper as it covers performance of an optimization algorithm, which we borrow from literature (any choice of the algorithm would have its (dis)advantages). However, one can design a synthetic case for comparison. Given the above we give such an example in this rebuttal letter.

We have performed optimization with up to ten data sets with 5 features and 300 samples. Each DATA scatter was drawn from a standard normal distribution N(0,1). MODEL function

generates data for each data set (again, 5 features and 300 samples) by varying two parameters of the standard normal distribution, i.e. N(x1,x2). We fix all other configurations of the optimization. The results are shown in the figure at https://github.com/inuritdino/BayesForest/wiki/Additional-data#optimization-efficiency-vs-number-of-data-sets .

Since we know the true values of x1 and x2 (0.0, and 1.0, respectively), we can assess the optimization performance as a function of number of data sets by calculating max and min deviations from the true values across the data sets in a series of similar optimization runs (6 runs per case). One can see from the figure that the median profile for the maximum deviation increases with number of data sets.

Note such increase in the estimate deviations is for the given optimization configuration. For example, we have verified that if we allow for larger number of iterations, we will not observe the increase (data not shown) as a function of number of data sets, since the optimization for any number of data sets between 1 and 10 is equally good given the longer search time. To observe the increase one would need to increase the number of data sets even more. Thus, it is purely a characteristic of the optimization routine.

8) Overall, this work is useful to the community, given that the authors have provided an open source platform. Some of the points that need clearer presentation are: efficiency of the algorithm (compared to other approaches), pros and cons of their current work, best case plant species where they anticipate this method to work, is it immediately usable or is it still work in progress, computational requirements for the end user. Some of this information may be available in the manuscript, but is qualitative and not quantitative as would be expected from an algorithmic approach.

- We have tried to address this issue by including a new section on the algorithm's performance.

9) Regarding usage of Matlab toolbox: After going through the Matlab code, some small modules are working but to replicate the results of the original paper will take 1 day of computing. Therefore, the authors have suggested to use the smaller test set. But there is no clear instruction of how to run the input-test.txt file.Specifically the authors should detail how to read a txt input file using the example of input-test.txt file, what does the test file actually represent and what output will be obtained upon running these. This will help potential users validate the code and its usefulness without having to simulate the results of the paper which are very time and compute intensive. Instructions on how to actually tweak some parameters and see changes would be handy for reproducibility. This would then encourage users to actually run heavy computation on the entire dataset.

- We have launched the new BayesForest Wiki website: https://github.com/inuritdino/BayesForest/wiki. These Wiki's have also a small tutorial explaining in detail how to run the two tests, what they do, what results are produced etc. Test 1 link: https://github.com/inuritdino/BayesForest/wiki/Simple-examples#example-1 Test 2 link: https://github.com/inuritdino/BayesForest/wiki/Simple-examples#example-2
