# Supplementary material for: Bayes Forest: a data-intensive generator of morphological tree clones
Source: Gigascience. 2017 Aug 19;6(10):1–13. doi: 10.1093/gigascience/gix079 (PMC5632294; doi:10.1093/gigascience/gix079)
Supplement: Reviewer-1-Report-(Original-Submission).pdf [file gix079_Reviewer-1-Report-(Original-Submission).pdf]

Reviewer Report

Title: Bayes Forest: a data-intensive generator of morphological tree clones

Version: Original Submission **Date:** 5/25/2017

Reviewer name: Jean-francois Cote

Reviewer Comments to Author:

The manuscript describes a tree modeling approach using a quantitative structural model (QSM) for reconstructing the intricate details of trees from terrestrial lidar data. Then, a stochastic growth model is first parameterized (through optimization) by comparing structure attributes from the QSM, and applied to reproduce a large amounts of similar trees or clones. The tree generator is quite innovative and could serve in many forest ecological studies where a variety of tree structure is required but with limited data on the actual trees under studies extracted from terrestrial lidar. Overall, the technical note is generally well written and the methods detailed with sufficient information. However, I found the section ordering a bit confusing. The methods section is found at the end of the manuscript, but many variables and concepts that are defined in this section are referred in the previous ones. This makes it difficult for the reader to follow the ideas and concepts explained without having to read ahead in the text. Depending on the editorial choice in the section ordering, moving the "methods" before "III. Testing for the algorithm" would certainly improve the reader experience. Another element which needs to be looked at is the extensive use of sub-sentence parts enclosed by parenthesis. It is generally not a good practice and breaks the text flow. Those should be either written as independent sentences or simply removed. Lastly, the manuscript has some grammar and language issues, which need to be addressed. Specific comments and recommendations can be found in the annotated PDF attached with this review report.

Level of Interest

Please indicate how interesting you found the manuscript: An article whose findings are important to those with closely related research interests

Quality of Written English

Please indicate the quality of language in the manuscript: Needs some language corrections before being published

Declaration of Competing Interests

Please complete a declaration of competing interests, considering the following questions:

- Have you in the past five years received reimbursements, fees, funding, or salary from an organisation that may in any way gain or lose financially from the publication of this manuscript, either now or in the future?
- Do you hold any stocks or shares in an organisation that may in any way gain or lose financially from the publication of this manuscript, either now or in the future?
- Do you hold or are you currently applying for any patents relating to the content of the manuscript?
- Have you received reimbursements, fees, funding, or salary from an organization that holds or has applied for patents relating to the content of the manuscript?
- Do you have any other financial competing interests?
- Do you have any non-financial competing interests in relation to this paper?

If you can answer no to all of the above, write 'I declare that I have no competing interests' below. If your reply is yes to any, please give details below.

I declare that I have no competing interests

I agree to the open peer review policy of the journal. I understand that my name will be included on my report to the authors and, if the manuscript is accepted for publication, my named report including any attachments I upload will be posted on the website along with the authors' responses. I agree for my report to be made available under an Open Access Creative Commons CC-BY license (http://creativecommons.org/licenses/by/4.0/). I understand that any comments which I do not wish to be included in my named report can be included as confidential comments to the editors, which will not be published.

I agree to the open peer review policy of the journal
