# Supplementary material for: Bayes Forest: a data-intensive generator of morphological tree clones
Source: Gigascience. 2017 Aug 19;6(10):1–13. doi: 10.1093/gigascience/gix079 (PMC5632294; doi:10.1093/gigascience/gix079)
Supplement: Reviewer-2-Report-(Original-Submission).pdf [file gix079_Reviewer-2-Report-(Original-Submission).pdf]

Reviewer Report

Title: Bayes Forest: a data-intensive generator of morphological tree clones

Version: Original Submission **Date:** 6/6/2017

Reviewer name: Srikant Srinivasan

Reviewer Comments to Author:

The concept and application of tree form generators is a valuable data scientific tool with a wide range of applications. The authors have adequately represented the past work leading to their current submission and emphasized their unique contributions. Although this work is in the context of plant sciences, there is not much plant science involved in it and it deals with the mathematics of shape reconstruction. Overall, such a resource would be a useful addition to the community if the results of this work have quantitative bearing on any specific application in plant related topics. Given the algorithmic emphasis of their article, I feel a few points need to be better presented to understand the applicability of their work:1) Ignoring the improvements in algorithm, how useful is the result of their Bayes-Forest (BF) from a plant science perspective? Have they been able to accurately emulate any plant characteristics that have resulted in better scientific understanding of plants.... or made things easier for plant phenotyping etc?2) Can the authors take an example tree and compare their outcome with the FSPM of the same? Can the BF accommodate external abiotic factors? If not what are the limits of its usefulness.3) Fig.1: Rework Fig. 1 to make the flow clearer. How is the empirical distribution Um obtained from the SSM? Give quantitative examples of stopping criteria for optimization. How are these thresholds chosen?4) Line 177: how the influential parameters quantitatively determined?5) Line 205 - 208: How are the relevant number of structural features determined? What is the scaling time/complexity and data size associated with these choices? provide a graph showing the relationship for a given plant type to give a better understanding.6) Fig. 5 shows significant difference from target QSM values for several features. Only height seems to get close to the target. How useful are these results? Also what is the simulation time and computing requirements to achieve these results?7) Provide a graph showing the scaling of optimization time/ efficiency as a function of the number of {Um,Ud} data sets.Overall, this work is useful to the community, given that the authors have provided an open source platform. Some of the points that need clearer presentation are: efficiency of the algorithm (compared to other approaches), pros and cons of their current work, best case plant species where they anticipate this method to work, is it immediately usable or is it still work in progress, computational requirements for the end user. Some of this information may be available in the manuscript, but is qualitative and not quantitative as would be expected from an algorithmic approach.Regarding usage of Matlab toolbox: After going through the Matlab code, some small modules are working but to replicate the results of the original paper will take 1 day of computing. Therefore, the authors have suggested to use the smaller test set. But there is no clear instruction of how to run the input-test.txt file.Specifically the authors should detail how to read a txt input file using the example of input-test.txt file, what does the test file actually represent and what output will be obtained upon running these. This will help potential users validate the code and its usefulness without having to

simulate the results of the paper which are very time and compute intensive. Instructions on how to actually tweak some parameters and see changes would be handy for reproducibility. This would then encourage users to actually run heavy computation on the entire dataset.

Level of Interest

Please indicate how interesting you found the manuscript: An article whose findings are important to those with closely related research interests

Quality of Written English

Please indicate the quality of language in the manuscript: Acceptable

Declaration of Competing Interests

Please complete a declaration of competing interests, considering the following questions:

- Have you in the past five years received reimbursements, fees, funding, or salary from an organisation that may in any way gain or lose financially from the publication of this manuscript, either now or in the future?
- Do you hold any stocks or shares in an organisation that may in any way gain or lose financially from the publication of this manuscript, either now or in the future?
- Do you hold or are you currently applying for any patents relating to the content of the manuscript?
- Have you received reimbursements, fees, funding, or salary from an organization that holds or has applied for patents relating to the content of the manuscript?
- Do you have any other financial competing interests?
- Do you have any non-financial competing interests in relation to this paper?

If you can answer no to all of the above, write 'I declare that I have no competing interests' below. If your reply is yes to any, please give details below.

'I declare that I have no competing interests'

I agree to the open peer review policy of the journal. I understand that my name will be included on my report to the authors and, if the manuscript is accepted for publication, my named report including any attachments I upload will be posted on the website along with the authors' responses. I agree for my report to be made available under an Open Access Creative Commons CC-BY license (http://creativecommons.org/licenses/by/4.0/). I understand that any comments which I do not wish to be included in my named report can be included as confidential comments to the editors, which will not be published.

I agree to the open peer review policy of the journal
